# Self-experienced empathetic behaviour patterns in medical students during virtual patient encounters: a comparison between an AI-enhanced social robot and a computer-based platform

**DOI:** 10.3389/frai.2026.1795842

**Published:** 2026-03-04

**Authors:** Alexander Borg, Benjamin Jobs, Cidem Gentline, Viking Huss, Anna Hugelius, Jonathan Schiött, William Ivegren, Fabricio Espinosa, Mini Ruiz, Samuel Edelbring, Carina Georg, Gabriel Skantze, Ioannis Parodis

**Affiliations:** 1 Division of Rheumatology, Department of Medicine Solna, Karolinska Institutet, Karolinska University Hospital, Center for Molecular Medicine (CMM), Stockholm, Sweden; 2 Division of Clinical Epidemiology, Department of Medicine Solna, Karolinska Institutet and Karolinska University Hospital, Stockholm, Sweden; 3 Department of Clinical Science, Intervention and Technology, Karolinska Institutet, Stockholm, Sweden; 4 Department of Educational Sciences and Arts, Faculty of Philosophy, Mälardalen University, Västerås, Sweden; 5 Unit of Teaching and Learning, Karolinska Institutet, Stockholm, Sweden; 6 School of Health Sciences, Örebro University, Örebro, Sweden; 7 Department of Neurobiology, Care Sciences and Society, Karolinska Institutet, Stockholm, Sweden; 8 Division of Speech Music and Hearing, Royal Institute of Technology (KTH), Stockholm, Sweden; 9 Department of Rheumatology, Faculty of Medicine and Health, Örebro University, Örebro, Sweden

**Keywords:** empathy, large language models, medical education, social robotics, virtual patients

## Abstract

**Objective:**

To explore whether an AI-enhanced social robotic virtual patient (VP) platform reinforces empathetic behaviour patterns in medical students compared with a traditional computer-based platform.

**Methods:**

Twenty-three sixth-semester medical students from Karolinska Institutet participated in semi-structured interviews following VP encounters with the Social AI-enhanced Robotic Interface (SARI) and, as a comparator, the computer-based Virtual Interactive Case system (VIC). Additionally, 178 students evaluated the VP platforms in empathetic training quantitatively using categorical nominal variables and a visual analogue scale (VAS), with a score of 0 indicating full preference for SARI and 10 full preference for VIC. Interview data were thematically analysed, and quantitative preferences were compared using the Fisher’s exact test with Monte Carlo simulation and the Wilcoxon signed-rank test.

**Results:**

Thematic analysis yielded five major themes wherein students consistently reported that SARI facilitated greater empathetic engagement through multimodal interaction, ability to express emotions, and real-time communication adaptability. Quantitative analysis demonstrated a higher preference for SARI versus VIC (78% versus 6%; OR: 190.4; 95% CI: 76.8–472.0; *p* < 0.001), which remained consistent across subgroups of interest, i.e., female and male students, with and without prior experience in VPs, and students first exposed to SARI or first exposed to VIC. VAS data also showed a preference for SARI versus VIC (median: 2.00; IQR: 1.00–4.00; *W*: 738.5; *r*: 0.70; *p* < 0.001).

**Conclusion:**

Our AI-enhanced social robotic VP platform was superior to a traditional computer-based VP platform in fostering empathetic engagement in medical students through enhanced authenticity and interactivity, supporting its potential to supplement clinical rotations.

## Highlights

- The Social AI-enhanced Robotic Interface (SARI) for virtual patient simulation creates a more authentic environment for medical students, fostering empathy and immersive learning.
- Medical students experienced that SARI reinforces empathetic engagement through multimodal and advanced ways of interaction.
- Large language model (LLM)-enhanced social robotic virtual patient platforms may prove useful as safe learning environments for developing empathetic communication skills, alongside training of clinical reasoning.

## Introduction

Empathy is by some considered one of the most important skills of healthcare practitioners who are engaged in patient care (Colliver et al., 2010). Empathetic skills from healthcare providers have been shown to be associated with better health outcomes for patients and decreased risk of hospitalisations (Del Canale et al., 2012; Hojat et al., 2011). Patient-perceived empathy from physicians has been shown to influence patient satisfaction, adherence, and interpersonal trust (Emamikia et al., 2022; Kim et al., 2004), which are important factors in improving health outcomes for patients. Due to its multidimensional nature, empathy has been challenging to define, leading to varied descriptions across the literature (Hojat, 2007). Within the field of medical education, empathy is mostly described using cognitive characteristics, such as being able to understand the perspective of a patient, in contrast to emotional characteristics, such as feeling a patient’s pain or suffering (Sulzer et al., 2016). Such emotional characteristics are more commonly associated within the concept of sympathy, which, however, can overlap with empathy (Hojat et al., 2001).

In undergraduate medical education, the cultivation of empathetic behaviour is commonly facilitated through structured interactive modalities. Role-play scenarios constitute a primary approach, either between peers or with trained actors serving as standardised patients. These standardised patients are instructed to simulate specific clinical conditions with predetermined behavioural patterns and emotional responses, enabling students to practise empathetic communication within controlled yet realistic settings. This approach ensures consistent exposure to diverse patient presentations while allowing students to develop empathetic responses in a safe learning environment before meeting real patients (Patel et al., 2019). However, standardised patients can be costly for educational sites, and availability aspects can further hurdle the training of empathetic conduct. An alternative to using standardised patients to practise empathetic conduct could be virtual patients (VPs).

VPs are digital educational modalities that are often used to resemble various clinical case scenarios, with the goal of practising and achieving specific learning outcomes in health professions education (HPE) curricula (Ellaway, 2006; Hege et al., 2016; Xu et al., 2023). VPs are acknowledged as a good complement to real-life patient encounters, with ability to ensure the practise of clinical skills, e.g., clinical reasoning, procedural, and team-based skills (Kononowicz et al., 2019). They have also been shown to be beneficial modalities in training empathetic communication in early stages of medical education by facilitating interactions in a low pressure environment (Kleinsmith et al., 2015). However, when compared with standardised patients, students’ interactions with VPs have been shown to generate less empathetic responses (Deladisma et al., 2007). Technological advancements have led to the emergence of artificial intelligence (AI) and large language models (LLMs), which enable more realistic VP interactions compared to traditional modalities (Cook, 2024; Suárez et al., 2022), potentially improving the possibilities of empathetic behaviour towards VPs. In previous work, we introduced a novel VP modality by applying an LLM in combination with a social robot. This was perceived by medical students as a more realistic and more authentic platform compared to a conventional computer-based VP platform (Borg et al., 2025; Borg et al., 2024). To our knowledge, there are no previous studies that investigate empathetic behaviour patterns towards VPs presented through an AI-enhanced social robot.

The aim of this study was to explore if an AI-enhanced social robotic VP platform can reinforce self-experienced empathetic behaviour in medical students compared with a traditional computer-based platform.

## Methods

We conducted an interventional explorative study to examine how medical students perceived their empathetic behaviour when using a social robotic VP platform enhanced with an LLM, compared to a conventional computer-based VP platform. The conventional computer-based platform virtual interactive case system (VIC) (Virtual Interactive Case System, n.d.) employs a semi-linear design structure where students navigate through predetermined pathways by selecting among questions and receiving fixed text-based responses. In contrast, our newly developed social AI-enhanced robotic interface (SARI) (Borg et al., 2025; Borg et al., 2024) enables branched conversations where each interaction is contextually generated based on specific queries and the conversation history. While VIC provides consistent, reproducible interactions through its structured format, SARI offers adaptive, naturalistic dialogue with multimodal communication including facial expressions, voice modulation, and real-time emotional responses (Huwendiek et al., 2009).

Data for this comparison were derived from qualitative fully transcribed in-depth interviews and a quantitative questionnaire for VP platform evaluation wherein students rated which platform they preferred for practising empathetic skills. The qualitative part of the study followed consolidated criteria for reporting qualitative research (COREQ) (Tong et al., 2007). The report is detailed in Supplementary Table S1.

### Participants

In this study, we recruited sixth-semester medical students from Karolinska Institutet (KI) in Stockholm, Sweden. The recruitment took place during the course “Clinical medicine 2: applied internal medicine and related disciplines,” specifically during clinical placements within rheumatology at the Karolinska University Hospital. The sixth semester was chosen as it marks the transition from pre-clinical to clinical education during the KI medical programme, including the first clinical rotations. This represents a formative period for empathy development, as medical students have acquired foundational medical knowledge but are at the beginning of regular patient contact. Participants in the in-depth interviews (*n* = 23) were recruited from a pool of students who undertook the course during the spring term of 2024 (*n* = 117). Of 60 students who consented to participate in interviews, 23 students were finally included, determined by the chronological order of the participant response. The number of interviews was considered sufficient as it yielded information power (Malterud et al., 2016) during thematic analysis, as per the COREQ recommendations (Tong et al., 2007). Furthermore, 178 students agreed to participate in quantitative evaluations of the VP platforms and were recruited between the spring term of 2024 and the spring term of 2025.

Participation in the study was voluntary, and all participants provided written informed consent prior to inclusion and could withdraw their participation at any time. The study was approved by the Swedish Ethical Review Authority prior to enrolment (registration number: 2022–04437-01).

### VP case development and practise

Ten VP cases were developed according to distinct recommendations for VP platform development (Posel et al., 2009). They were designed to be in English to also facilitate accessibility for international students. The cases were presented in two VP platforms: our newly developed SARI and the computer-based interface VIC (Virtual Interactive Case System, n.d.). Each platform contained five cases, with one having identical case content between the two platforms (Borg et al., 2025). The case development followed distinct principles and guidelines for development of VP cases (Posel et al., 2015), and used clinical case content illustrating various rheumatic conditions, as previously described by our group (Borg et al., 2025; Borg et al., 2024).

Students performed VP cases in pairs or groups of three to promote collaboration, based on previous work that has demonstrated enhanced CR training (Edelbring et al., 2018). The cases started with a small introduction of a patient case within a specific setting, and students completed each VP case when they perceived that they had sufficient information for concluding to a preliminary diagnosis and a management plan. Since SARI primarily communicated using its voice, students were provided written information before the VP encounters based on the context of each case, as well as results from relevant laboratory tests along with their corresponding reference values. Following each completed case, students participated in follow-up seminars facilitated by a physician specialised in rheumatology to discuss the case contents. The seminar groups ranged between six and eight student participants. Half of the students started VP practise with SARI and half of the students started with VIC. All students completed all VP cases and participated in all follow-up seminars during their clinical placement.

### The social robotic platform SARI

For the embodiment of SARI, we used the social robot developed by Furhat robotics, which has an animated face that matches the patients’ age and sex, projected on a semi-transparent plastic face mask, and a mechanical neck that allows natural head movements (Al Moubayed et al., 2012). Furhat displays subtle facial expressions and affective responses, including gaze behaviour as indicators of a patient’s emotional status (Mishra et al., 2023). Furhat includes the Furhat software development kit (FurhatSDK) (Al Moubayed et al., 2012), which we combined with the Open AI gpt-3.5 turbo LLM (Brown et al., 2020) to develop SARI. We prompted the LLM to generate authentic dialogue responses from the perspective of a patient using specific instructions and included a detailed patient description together with the last 10 dialogue lines. To reflect the emotional state of a patient, we prompted the LLM to generate suitable facial expressions during the conversation using anchor points (Irfan et al., 2023). Appropriate facial expressions were then presented at these anchor points by the social robot from a predefined set of facial expressions in the FurhatSDK. The selection of facial expressions was based on the context of the dialogue and the LLM-generated responses from the VP. An example of a prompt is illustrated in the Supplementary Figure S2.

A known limitation of LLMs and social robots in turn-taking dialogue is response delays (Irfan et al., 2023). To mitigate the risk of misunderstanding due to response delays, we created a turn-taking signal using a LED-light at the bottom of the robot to signal if the robot was actively listening or preparing a response using specified colours (Skantze et al., 2015).

### The computer-based platform VIC

VIC is a computer-based VP platform where users can explore patient case details in any order they choose, while the introduction and conclusion remain fixed (Virtual Interactive Case System, n.d.). Users gather information by selecting pre-written questions about the patient’s medical history and are instructed to gather relevant information within the context of the case. They can also conduct virtual physical examinations and review test results and reports from relevant imaging. When students felt they had collected sufficient information, they completed the case by diagnosing the condition and creating a management plan for the VP from a selection of multiple-choice options (Ahn and Edelbring, 2020; Georg and Zary, 2014).

### Data collection

Following completion of all VP cases in both SARI and VIC as well as the follow-up seminars, students who consented to participate in the study evaluated the VP platforms quantitatively by responding to two questions regarding the VP encounter about empathetic conduct. The first question was “overall, which of the platforms is preferable to you in relation to self-experienced empathy during the patient encounter?”, which was answered using one of three options: (i) SARI, (ii) VIC, or (iii) equally preferred. The second question followed the structure of a visual analogue scale (VAS) and read “on a scale between 0 and 10, where 0 indicates total preference of the social robot and 10 indicates total preference of the computer-based platform, how would you grade your preference of the virtual patient platforms compared to each other in relation to self-experienced empathy during the patient encounter?”; on this scale, 5 indicated equal preference between the two platforms. Students also provided additional data such as age, sex, whether they had previous experience with VP platforms, and which platform they were introduced to first.

Some students also participated in semi-structured interviews. The interviews followed an interview guide that pertained learning experiences, the platform contribution to acquirement of communication skills, self-perceived empathy, as well as suggestions for improvements. The interview guide was an iteration of the guide used for the pilot testing of SARI (Borg et al., 2025), following refinements based on feedback provided by medical students. The interview guide is provided in Supplementary Figure S3.

All interviews were conducted by the same researcher (AB), either in person at the Karolinska University Hospital or during video calls. Each interview ranged between 40 and 60 min, was fully recorded, transcribed verbatim, and pseudonymised. Interview data were stored on secure servers at KI and were accessible only to involved researchers upon removal of personal or sensitive information.

### Interview analysis

We processed fully transcribed interview data systematically according to the six-phase methodological approach for reflexive thematic analysis described by Clarke and Braun (Braun and Clarke, 2006; Braun and Clarke, 2021; Braun and Clarke, 2023). During analysis and theme development, two researchers (AB and BJ) were perceptive to the emergence of themes to capture unique aspects of the students’ experiences.

Firstly, researchers (AB and BJ) independently read and re-read all transcripts to note initial ideas and patterns related to empathetic behaviour. Secondly, codes were implemented systematically across the entire dataset, connecting relevant data to each code. The codes included specific references to emotional responses, communication patterns, and platform interactions. Thirdly, initial themes emerged by the connection of codes into overarching categories, gathering all relevant data to each potential theme. Fourthly, the themes were reviewed against coded extracts multiple times. This involved ensuring themes were internally coherent and distinct from each other. Fifthly, the final themes were defined and named to generate clear definitions and names that capture the essence of each theme. Finally, a report was produced by selection of representative quotes from the transcripts and final analysis relating back to the research question on empathetic behaviour towards VPs.

The coding process was collaborative and iterative. Researchers (AB and BJ) met regularly on a weekly basis to compare and discuss their interpretations, emerging patterns, and refine the coding framework. Following discussions, the codes were refined to resolve any discrepancies until the coding scheme resulted in consensus on the final thematic structure. The codes were used in specific areas of the interviews to highlight aspects of empathetic behaviour towards VPs. The analysis, coding process, and theme development were undertaken by AB and BJ, with IP providing supervision and guidance on the final thematic structure.

### Statistics

The Fisher’s exact test with Monte Carlo simulation (10,000 iterations) was used to compare frequencies of categorical responses on VP platform preference for self-experienced empathy. The Wilcoxon signed-rank test was used for VAS-scale responses comparing the scores versus a hypothetical neutral score of 5. Results from Fisher’s exact tests are presented as numbers and the corresponding percentage, odds ratio (OR), 95% confidence interval (CI), and *p* value. Results from Wilcoxon signed-rank tests are presented as the median and the corresponding interquartile range (IQR), test statistic (*W*), effect size (*r*), and *p* value. The statistical analysis was performed using R software, version 4.3.3 (R foundation for Statistical Computing, Vienna, Austria). Differences yielding *p* values <0.05 were considered statistically significant.

## Results

### Interviews

Of 23 students who participated in interviews, 61% were men (*n* = 14) and 39% were women (*n* = 9). A total of 74% had no previous experience with VPs (*n* = 17). The students’ mean age was 23.5 (SD: 6.0) years. Regarding order of platform interaction, 52% started with SARI (*n* = 12), whereas 48% started with VIC (*n* = 11). Thematic analysis resulted in five themes: (i) physical embodiment, (ii) responses to emotional cues, (iii) cognitive immersion, (iv) empathetic interaction, and (v) complementary learning value. Each theme was further divided into sub-themes, totalling eight sub-themes across all themes. Table 1 details the identified themes along with their corresponding sub-themes and analytical codes. Illustrative quotes from students are shown in Table 2. Examples of the reflexive analysis process are provided in Supplementary Table S4.

**Table 1:** Identified themes and sub-themes from the qualitative thematic analysis.

Themes	Sub-themes	Analytical codes
Physical embodiment	Multimodal communication	Visual/facial expressions
		Verbal interaction quality
		Interactive experience quality
	From text to embodied interaction	Perception of patient authenticity
		Emotional responses to VPs
		Presence of emotional connection
Responses to emotional cues	Responding to concerns	Emotional responses to VPs
		Sense of responsibility
		Ability to express comfort/reassurance
	Adaptability of communication	Interactive experience quality
		Verbal interaction quality
		Ability to express comfort/reassurance
Cognitive immersion	Active engagement	Differences in question formulation
		Interactive experience quality
		Learning focus
	Memory and continuity	Interactive experience quality
		Verbal interaction quality
		Perception of patient authenticity
Empathetic interaction		Emotional connection
		Sense of responsibility
		Emotional responses to VPs
		Perception of patient authenticity
Complementary learning value	Clinical reasoning versus empathy	Learning focus
		Interactive experience
		Ability to express comfort/reassurance
	Standardisation and variance	Interactive experience
		Differences in question formulation
		Learning focus

**Table 2:** Relevant quotes from in-depth interviews by theme and sub-theme.

Study participant	Quote
Physical embodiment	
Multimodal communication	
Female student, 21 years old	“It was the facial expressions. You could hear it in the voice. ‘What can it be? I’m worried. I’ve had so many complaints.’ And then you had to face it.”
Male student, 21 years old	“The face adds quite a lot because you have someone to look at. Even if it’s very difficult to get an emotional connection, the little you get comes from the face. Without it, you get zero, I’d say.”
From text to embodied interaction	
Female student, 21 years old	“The computer was like a game. And like a text that you read. So I did not feel close to the patient at all. But for the robot, when it talked, it was still like a real patient.”
Male student, 21 years old	“Yes, but I think it was easier to feel empathy for the robot cases. Because they talk. They express what they think is tough. And then it’s more like you can get into it.”
Male student, 42 years old	“When patients talk to me, I try to picture what they describe […] when I got this told in speech by this [robotic] virtual patient, and also in some cases facial expressions and so on, I feel that I live in the patient’s situation in a completely different way than when I read a text.
Responses to emotional cues	
Responding to concerns	
Female student, 21 years old	“When it started to express feelings, […], it was like my brain started to understand that this is almost a real person.”
Female student, 21 years old	“And when it expressed its worries, you were like, ‘I want to answer its worries so that it does not walk around and get anxious.’ So I felt closer to the patient.”
Male student, 22 years old	“Sometimes when it was worried about something and so on, you still felt that you wanted to answer its worries. And we did it like this several times. We tried to calm down the patient.”
Adaptability of communication	
Male student, 25 years old	“When you talked to the robot it affected a part of you. You confirm, mirror, and summarise, so you feel more empathetic.”
Male student, 21 years old	“With the social robot, you lose that [stress] and can focus on learning, which will help you to encounter patients. But you still get that human connection, where it feels more real.”
Cognitive immersion	
Active engagement	
Male student, 42 years old	“The robot, the face-to-face conversation, and getting a real response to the questions I asked, that created a lot of immersion.”
Male student, 21 years old	“I’m not an expert in neurology, but if I just try to think about it, it does not feel like it’s the same parts that are activated by writing a question and formulating it, like actually sitting and formulating it in front of someone. You cannot stop halfway through a sentence, erase half of it and rewrite it when you start talking to someone. In a way, you really need to have a good idea of what you are going to say before you say it.”
Memory and continuity	
Male student, 21 years old	“It was a conversation where you got to remember the answers you had received earlier while you were thinking about the next question. So it felt closer to reality in a way.”
Female student, 23 years old	“I think that these robot cases […] you are forced to be more active. And then I also experienced that you remember more afterwards. […]”
Empathetic interaction	
Female student, 22 years old	“The robots were more authentic [in allowing empathetic behaviour]. It felt more like an interaction with a patient. While the computer-based platform […] it’s more restricted. It’s not a patient that you can see.”
Female student, 21 years old	“It [the robot] answers your questions and can express feelings. It builds on […] authenticity.”
Complementary learning value	
Clinical reasoning versus empathy	
Male student, 21 years old	“The advantages are that the virtual patient cases are more straightforward. Real patients can often be very complicated. They often have a lot. It can be difficult to find out what the real problem is.”
Male student, 42 yeasr old	“Learning-wise, I cannot speak for the others, but I think I became more committed and took it to an even higher degree. I try to learn with all the opportunities I get, but I think it is the commitment, that you become more committed in the robotic case, plus, as I said earlier, you are forced to formulate things.”
Standardisation and variance	
Female student, 48 years old	“And then also, because it is a virtual patient case, there is also the possibility that everyone should get the same patient, and that you should also be able to get the same, the most important and most common diseases.”
Female student, 23 years old	“One thing I thought about with the robotic cases. […] Different groups received different information. This […] affects how you ask questions. […] with the computer-based cases you always get the same information.”

### Physical embodiment

The theme “physical embodiment” suggested that SARI’s physical presence influenced empathetic engagement compared to text-based interactions. This theme was further divided into two sub-themes, i.e., (i) multimodal communication and (ii) from text to embodied interaction.

In the subtheme “multimodal communication,” students consistently reported that the multimodal interaction through a combination of voice, facial expressions, and interactive responses from the robot created a more authentic experience compared with VIC, which facilitated empathetic engagement. The ability of SARI to convey emotional states through facial animations while speaking created a more nuanced communication experience. Students reported that witnessing worried expressions together with concerned vocal tones made them more likely to respond empathetically than when reading similar concerns in text.

In the subtheme “from text to embodied interaction,” the analysis revealed a perception shift when students transitioned from text-based to embodied VP interactions. Students described that VIC felt game-like and abstract, creating psychological distance towards the VP. In contrast, SARI transformed their perception of the VP from an educational exercise to an encounter with a quasi-person, which they perceived as stimulating towards empathetic engagement.

### Responses to emotional cues

The theme “responses to emotional cues” suggested that students recognised and responded to emotional expressions from the VPs, with distinct differences between the platforms. This theme was further divided into two sub-themes, i.e., (i) responding to concerns and (ii) adaptability of communication.

In the subtheme “responding to concerns,” students demonstrated higher awareness and responsiveness when interacting with SARI compared to VIC. When the robotic patient expressed worries or fear, students reported feeling compelled to respond to these emotional needs directly, resembling interactions with real patients. Students experienced a sense of responsibility for the robotic VP’s emotional state, prompting them to offer spontaneous reassurance and empathetic acknowledgment. This contrasted their approach to VIC, where emotional content was noted cognitively but did not evoke the same immediate empathetic response.

The subtheme “adaptability of communication” explored the interactive nature of the responses received from SARI, which required students to adapt their communication style based on those responses, fostering empathetic behaviour. Students reported adapting their questioning technique, tone, and pace in response to perceived patient distress or confusion. This adaptive process included using communication strategies typically used in real clinical encounters, such as mirroring, summarising, and validating. Such adaptive processes were not identified or discussed in the context of VIC.

### Cognitive immersion

The theme “cognitive immersion” captured how different levels of cognitive engagement between platforms influenced students’ capacity for empathetic connection. This theme was further divided into two sub-themes, i.e., (i) active engagement, and (ii) memory and continuity.

In the subtheme “active engagement,” the need to actively formulate questions and wait for responses created a more engaging and authentic learning experience compared to VIC. Students described that SARI required them to maintain continuity in the conversation and remember previous information from the consultation, resembling the cognitive demand of real-life clinical encounters.

In the subtheme “memory and continuity,” students reported that the conversational nature of SARI interactions required them to maintain continuity throughout the VP encounter, relying on working memory rather than visual references. The verbal communication made them more prone to remember details from the consultation, promoting active listening and mental organisation, compared to being able to repeatedly return to relevant information on a computer screen, as was possible in VIC.

### Empathetic interaction

The theme “empathetic interaction” captured students’ direct experiences and reflections on empathetic engagement with VPs in both platforms. Unlike the other themes, this emerged as a unified concept without being divided into distinct sub-themes.

Students reported experiencing varying degrees of empathy in their interactions with the two platforms, with SARI consistently evoking more empathetic responses compared to VIC. The enhanced authenticity of the multimodal social robotic interactions created conditions where students could practise empathetic communication more naturally. However, while they acknowledged that none of the two platform fully replicated the empathetic connection that becomes possible with real patients, students identified SARI as substantially closer to authentic clinical encounters in its capacity to evoke and allow practise of empathetic behaviours. The perceived authenticity of the interaction influenced the degree of empathy in students’ responses, with greater perceived authenticity leading to a higher degree of empathy.

### Complementary learning value

The theme “complementary learning value” recognised that both platforms offered distinct educational benefits, with their differences creating complementary learning opportunities. This theme was further divided into two sub-themes, i.e., (i) diagnostic versus empathetic skills, and (ii) standardisation and variance.

In the subtheme “diagnostic versus empathetic skills,” students identified different learning areas between the platforms. The structured format and comprehensive information in VIC optimised diagnostic reasoning training, allowing systematic exploration of clinical data without the interpersonal aspects. In contrast, SARI was seen as stronger in balancing diagnostic reasoning with empathetic communication, requiring students to obtain clinical information through interaction that evoked empathetic behaviour.

In the subtheme “standardisation and variance,” the two platforms offered distinct advantages in terms of standardised learning experiences. VIC ensured that all students received identical information, facilitated standardised assessment, and provided consistent representation of patient cases. In contrast, SARI allowed a controlled variance based on individual communication approaches, resulting in different groups obtaining slightly different sets of information. The students appreciated this variety in information as it stimulated rich discussions during follow-up seminars, where groups compared their different interaction strategies and the information they had gathered with the other student groups.

### Quantitative comparisons of self-perceived empathetic conduct

**Figure 1:**
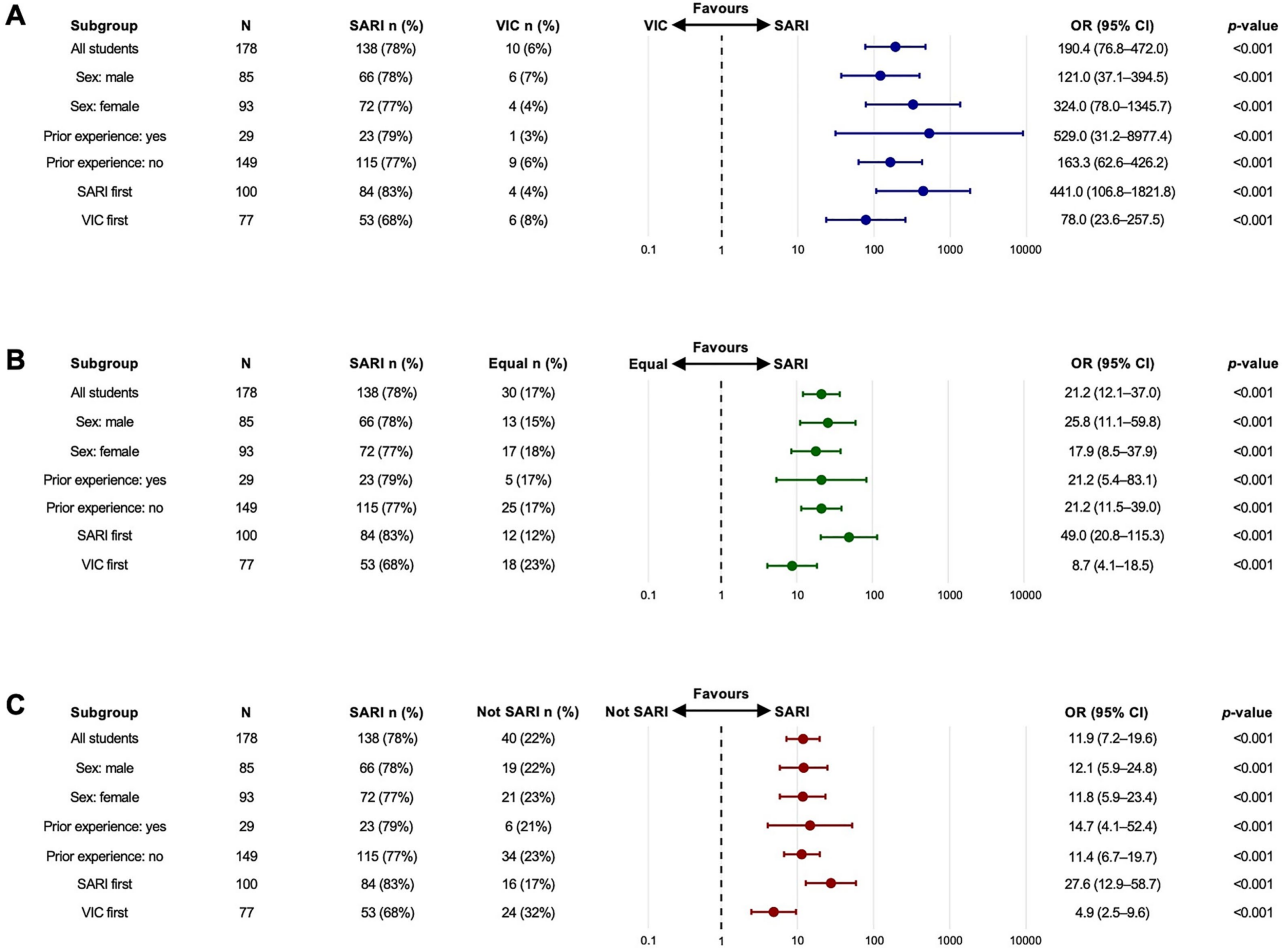
Forest plots illustrating results from Fisher’s exact test with Monte Carlo simulation on proportion of students preferring SARI versus comparator. Panel **(A)** shows comparisons between SARI and VIC (blue colour). Panel **(B)** illustrates comparisons between SARI and Equal preference (green colour). Panel **(C)** shows comparisons between SARI and Not SARI (red colour). Circles denote odds ratios (ORs) and whiskers 95% confidence intervals (CIs) on a logarithmic scale. SARI, Social AI-enhanced Robotic Interface; VIC, Virtual Interactive Case system.

**Figure 2:**
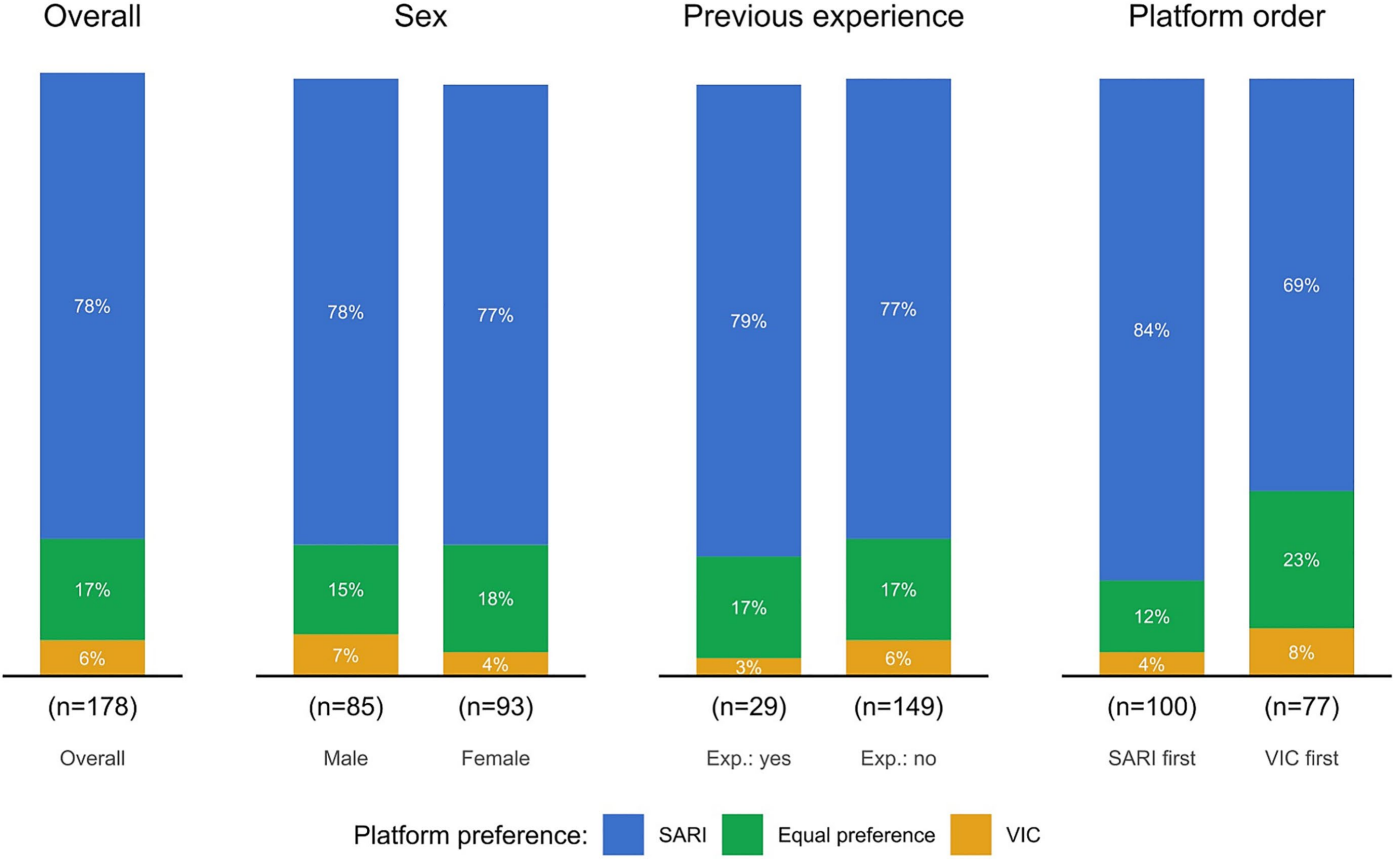
Bar plots illustrating the proportions of medical students’ self-perceived VP platform preference for empathetic conduct categorized by subgroups of interest (sex, previous experience of VP platforms, and platform introduced first). Exp., Experience; SARI, Social AI-enhanced Robotic Interface; VIC, Virtual Interactive Case system; VP, virtual patient.

Of 178 students who participated in quantitative evaluations, 93 (52%) were women and 86 students (48%) were men. Regarding prior exposure, 29 students (16%) reported previous experience with VPs, whereas 150 students (84%) had no previous experience. The students’ mean age was 25.3 (SD: 5.4) years. Regarding the order of platforms, 101 students (56%) started with SARI and 77 (43%) started with VIC. In relation to self-experienced empathy during the VP encounter, students preferred SARI compared to VIC (78% versus 6%; OR 190.4; 95% CI: 76.8–472.0; *p* < 0.001), compared to equal preference (78% versus 17%; OR: 21.2; 95% CI: 12.1–37.0; *p* < 0.001), and compared to VIC combined with equal preference (78% versus 22%; OR: 11.9; 95% CI: 7.2–19.6; *p* < 0.001). Similar patterns were seen after stratification into subgroups of interest, i.e., female or male students, students who had and students who did not have prior experience with VPs, as well as students who had started with SARI and student who had started with VIC. Results are illustrated in Figures 1, 2.

**Figure 3:**
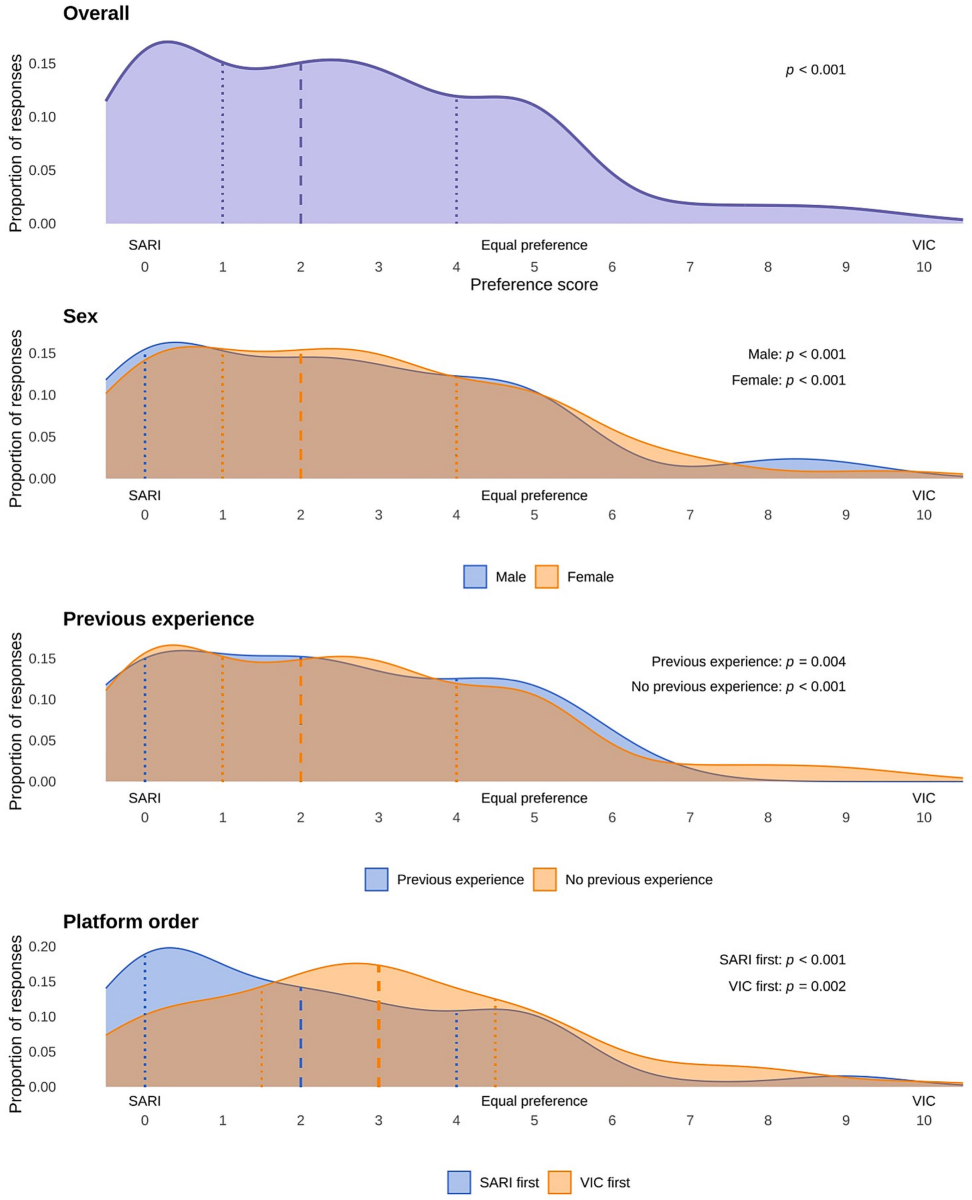
Density plots illustrating distributions of responses regarding VP platform preference. Shown are also results from Wilcoxon signed rank tests performed for comparisons of scores with a hypothetical score of 5 (equal preference of platforms) for each student. The different horizontal panels show, from top to bottom, the overall distribution of responses in the entire cohort of students, overlayed distributions in women and men, overlayed distributions in students with and without prior experience of VPs, and overlayed distributions in subgroups of students starting with SARI or VIC. SARI, Social AI-enhanced Robotic Interface; VIC, Virtual Interactive Case system; VP, virtual patient.

Results from VAS data demonstrated that students preferred SARI compared to VIC (median: 2.0; IQR: 1.0–4.0; *W*: 738.5; *r*: 0.70; *p* < 0.001). This difference remained statistically significant in comparisons within the student subgroups of interest mentioned above. Results are illustrated in Figure 3 and detailed in Supplementary Tables S5–S8.

## Discussion

Our study combined qualitative and quantitative methodology to investigate whether an AI-enhanced social robotic VP platform could foster a higher degree of self-experienced empathetic behaviour in medical students compared with a computer-based platform. The findings demonstrate a clear advantage of SARI over the conventional computer-based platform VIC for facilitating empathetic engagement. Thematic analysis of qualitative data yielded five major themes relating to empathetic behaviour consistently favouring SARI, while quantitative analysis confirmed these findings with students demonstrating a significant preference for SARI across all subgroups of interest.

The students’ experiences with SARI can be understood through the dual-process framework that distinguishes between cognitive and emotional empathy (Hojat, 2007; Sulzer et al., 2016). SARI appeared to promote cognitive empathy through requirements for active questioning, real-time interpretation of responses, and continuous dialogue. The themes “cognitive immersion” and its sub-themes “active engagement” and “memory and continuity” captured how students needed to actively generate questions and process information, mirroring the cognitive demands of real patient encounters. This aligns with the conceptualisation of empathy as a predominantly cognitive skill, as described by Sulzer et al. (2016), where understanding a patient’s perspective takes precedence over emotional mirroring. This stands in contrast to the more passive engagement observed with VIC, where pre-written questions limit the cognitive processes that are necessary for empathetic conduct.

Simultaneously, SARI appeared to promote emotional empathy through its multimodal expression capabilities. The themes “physical embodiment” and “responses to emotional cues” illustrate how students experienced spontaneous emotional responses when the robotic VP expressed worry or distress, making the students feeling compelled to address the VP’s emotional state and offer reassurance, suggesting that the multimodal interaction triggered affective empathetic processes beyond cognitive perspective-taking. The interplay between these two dimensions was particularly evident in the theme “empathetic interaction,” where students described how the enhanced authenticity of the multimodal interaction created conditions for a more engaging empathetic experience. The findings thus support the notion that empathy in medical education is not merely about emotional resonance but about active cognitive engagement with patient perspectives, a process that appeared to be better facilitated through the embodied interaction with the social robot and was seen as an important aspect of person-cantered communication (American Geriatrics Society Expert Panel on Person‐Centered Care et al., 2016; Hashim, 2017).

The advantages of SARI for fostering empathetic behaviour remained consistent despite individual characteristics and prior exposures, as evidenced by the consistent preference across all subgroups of interest. Contrary to previous literature suggesting variances in expressions of empathy between sexes (Berg et al., 2011; Hojat et al., 2002), our findings showed similar patterns between female and male medical students. Despite a numerical trend towards more students starting with SARI, suggesting a possible primacy effect, the platform order did not significantly influence the students’ preference. Similarly, prior VP experience did not substantially alter students’ preferences. This robustness across subgroups of interest strengthens our conclusion that the multimodal and interactive nature of SARI provides fundamental advantages for empathetic conduct that appeal broadly to medical students regardless of background characteristics.

Our findings align with emerging evidence on the value of physically embodied AI systems in healthcare education. A recent study showed that medical students who performed role-play with AI-enhanced humanoid robots for training English for medical purposes achieved significantly greater communication competence and empathy compared to those using only LLM-based virtual agents in a learning-by-teaching context (Derakhshan et al., 2025). Taken together, these observations suggest that physical embodiment plays a crucial role in facilitating empathetic engagement. Furthermore, our results support previous work illustrating the role of a physical presence and multimodal interaction to facilitate deeper emotional engagement in dialogue (Cummings and Bailenson, 2016; Lee, 2004). SARI created conditions that allowed students to engage in empathetic behaviour with VPs through its ability to express and respond to emotions through facial expressions, voice modulation, and real-time communication adaptability—features that consistently enhanced the authenticity of the interaction and thereby supported empathetic responses. Our research team has previously demonstrated that this enhanced authenticity of SARI also positively impacts clinical reasoning training in medical students (Borg et al., 2025; Borg et al., 2024).

The integration of LLM-enhanced social robots into medical education raises important considerations about the broader role of AI in healthcare training and practise (Alam et al., 2023). As students increasingly interact with AI systems during their education, educators must consider how these experiences shape students’ approaches to person-centred care (Nagi et al., 2023). The positive perception of the social robotic platform suggests that, rather than detracting from humanistic aspects of medicine, thoughtfully designed AI systems may enhance students’ capacity for empathetic communication by providing safe environments for practise (Park and Whang, 2022). However, educators must also remain vigilant about the values and communication patterns being modelled by these systems, ensuring they align with best practises in person-centred care. As AI systems become more prevalent in medicine, early exposure to such technologies through educational platforms like SARI may help students develop appropriate levels of critical engagement with AI-generated information (Ali, 2025). Importantly, AI-enhanced tools like SARI should be viewed as complementary for empathy training rather than replacements of human encounters. While our findings demonstrate that SARI can effectively facilitate both cognitive and emotional dimensions of empathetic engagement, the irreplaceable elements of genuine human connection must remain central to clinical education. The role of such platforms is therefore to provide accessible opportunities for students to develop and practise empathetic communication skills in a safe environment, thereby better preparing them for the humanistic demands of real clinical encounters.

### Limitations and future directions

Despite these promising findings, our study design has several important limitations that should be considered when interpreting the results. The use of English rather than Swedish for the VP cases represents a constraint with potential impact on empathetic expression. Research has shown that communication of empathy can differ when using one’s native versus second language, with potentially reduced emotional resonance when using a non-native language (Caldwell-Harris, 2014). The students’ abilities to demonstrate empathetic communication may have been constrained compared to what they could have achieved in Swedish, potentially influencing our findings, although this limitation likely affected both platforms similarly. Research on bilingual emotional expression suggests that communicating in a second language can reduce emotional intensity and the naturalness of affective expression (Caldwell-Harris, 2014; Keysar et al., 2012), which may have constrained the depth of empathetic expressions on both platforms. While the differences we observed between SARI and VIC may represent conservative estimates of the actual differences, the fact that SARI still demonstrated significant advantages with high odds ratios despite this shared constraint speaks to the robustness of practising empathetic conduct via the embodied multimodal interaction of SARI compared to the click-based interaction in VIC. Furthermore, while the LLM-enhanced social robot represents an advancement in VP technology, it still faces technical challenges such as occasional mechanical responses and connection difficulties that could impact educational outcomes. Also, as SARI did not allow for physical examination options, this might have affected students’ perception of empathy. Due to physical touch being an important aspect of experiencing empathy in physician-patient relationships, inclusion of real physical examination options might have increased the empathetic immersion (Kelly et al., 2020). While SARI appeared robust in promoting empathetic engagement through verbal and visual modalities, future iterations that incorporate haptic feedback or tactile interaction options could further enhance the empathetic immersion and bridge this gap.

Despite these technological constraints, we aimed to mitigate methodological limitations by enrolling a relatively large sample size (*n* = 23) for the qualitative component, generating a rich and diverse dataset that increases the representativeness of our findings. The quantitative validation of platform preference with 178 students further strengthens our conclusions. However, it is important to note that the results from this study might not be translatable within other contexts, warranting further evaluations in different settings. Our quantitative evaluation relied on students’ self-perceived platform preferences rather than objective assessments. While this was a deliberate methodological choice, the absence of external measures such as validated empathy scales, observer-rated assessments, or behavioural coding represents a limitation. Future studies would benefit from incorporating objective assessment such as the Jefferson Scale of Empathy (Hojat et al., 2001), observer-rated assessment, or behavioural coding of student-VP interactions, to triangulate with self-reported data.

The transferability of empathetic skills developed during VP simulations to real patient encounters remains an open question that warrants investigation. In future research, it would be of interest to investigate this transfer through longitudinal studies with pre- and post-intervention assessments using validated empathy instruments and investigation of dose–response relationships between the number and frequency of VP training sessions and empathetic outcomes, such as observer-rated assessment or patient satisfaction data. Additionally, investigating how specific features of the social robotic interface of SARI (facial expressions, voice qualities, response timing) specifically contribute to empathetic engagement could help optimise future VP platforms. Lastly, exploring how cultural and linguistic factors influence empathetic responses to social robotic VPs could address important questions about the cross-cultural applicability of such educational technologies and inform adaptations needed across educational contexts.

### Concluding remarks

In summary, this study demonstrates that our LLM-enhanced social robotic VP platform offers substantial advantages over an established conventional computer-based VP platform for fostering empathetic behaviour in medical students. The physical embodiment, multimodal interaction, and responsive dialogue capabilities of SARI created conditions more conducive to empathetic engagement, as evidenced by both qualitative themes and quantitative preference data. These findings were consistent across student subgroups of interest, suggesting broad applicability. Despite technological limitations, the enhanced authenticity and interactivity conferred from the social robotic platform created a more engaging learning environment that bridges the gap between text-based simulation and real clinical encounters. As medical education evolves alongside technological advances, platforms like SARI may play an important role in complementing clinical rotations by providing standardised, accessible patient encounters, which help students acquire the crucial empathetic communication skills that are necessary for safe, efficient, and person-centred patient care, alongside training in clinical reasoning.

## Data Availability

The raw data supporting the conclusions of this article will be made available by the authors without undue reservation.
