# Supplementary material for: Self-experienced empathetic behaviour patterns in medical students during virtual patient encounters: a comparison between an AI-enhanced social robot and a computer-based platform
Source: Front Artif Intell. 2026 Mar 4;9:1795842. doi: 10.3389/frai.2026.1795842 (PMC12996069; doi:10.3389/frai.2026.1795842)
Supplement: Supplementary file 1 [file Data_Sheet_1.PDF]

Self-experienced empathetic behaviour patterns in medical students during virtual patient encounters: a comparison between an AI-enhanced social robot and a computer-based platform

SUPPLEMENTARY MATERIAL

TABLE OF CONTENTS

SUPPLEMENTARY TABLE S1. CONSOLIDATED CRITERIA FOR REPORTING QUALITATIVE RESEARCH (COREQ): A 32-ITEM CHECKLIST FOR INTERVIEWS AND FOCUS GROUPS. 3
SUPPLEMENTARY FIGURE S2. EXAMPLE OF PROMPT USED FOR THE SOCIAL AI-ENHANCED ROBOTIC INTERFACE (SARI). 5
SUPPLEMENTARY FIGURE S3. INTERVIEW GUIDE EXPLORING STUDENTS' PERCEPTION OF VIRTUAL PATIENT PLATFORMS REGARDING EMPATHETIC CONDUCT. 6
SUPPLEMENTARY TABLE S4. EXAMPLES OF THE REFLEXIVE ANALYSIS PROCESS. 9
SUPPLEMENTARY TABLE S5. OVERALL WILCOXON SIGNED-RANK TEST RESULTS FROM VAS DATA ON STUDENT PREFERENCE OF VP PLATFORMS. 10
SUPPLEMENTARY TABLE S6. WILCOXON SIGNED-RANK TEST RESULTS FROM VAS DATA ON STUDENT PREFERENCE OF VP PLATFORMS BASED ON DEMOGRAPHIC SUBGROUP: SEX. 11
SUPPLEMENTARY TABLE S7. WILCOXON SIGNED-RANK TEST RESULTS FROM VAS DATA ON STUDENT PREFERENCE OF VP PLATFORMS BASED ON DEMOGRAPHIC SUBGROUP: PREVIOUS EXPERIENCE. 12
SUPPLEMENTARY TABLE S8. WILCOXON SIGNED-RANK TEST RESULTS FROM VAS DATA ON STUDENT PREFERENCE OF VP PLATFORMS BASED ON DEMOGRAPHIC SUBGROUP: PLATFORM ORDER. 13

Supplementary Table S1. Consolidated criteria for reporting qualitative research (COREQ): a 32-item checklist for interviews and focus groups.

Item	Description
**Domain 1: Research team and reflexivity**	
Personal Characteristics	
1. Interviewer/facilitator	Alexander Borg
2. Credentials	MD; PhD student
3. Occupation	Research intern
4. Gender	Male
5. Experience and training	Experience from PhD courses and supervision from supervisors
Relationship with participants	
6. Relationship established	None
7. Participant knowledge of the interviewer	None
8. Interviewer characteristics	Interest in pedagogical studies and teaching
**Domain 2: Study desing**	
Theoretical framework	
9. Methodological orientation and Theory	Phenomenology
Participant selection	
10. Sampling	Consecutive selection
11. Method of approach	Face-to-face
12. Sample size	23
13. Non-participation	94. Stated reasons were not required.
Setting	
14. Setting of data collection	Clinic

15. Presence of non-participants	None
16. Description of sample	Demographic data, date
Data collection	
17. Interview guide	Used and pilot tested
18. Repeat interviews	None.
19. Audio/visual recording	Audio recording was used
20. Field notes	Field notes were made after the interviews
21. Duration	40–60 minutes
22. Data saturation	Data saturation was discussed after interviews
23. Transcripts returned	Not returned
**Domain 3: analysis and findings**	
Data analysis	
24. Number of data coders	3
25. Description of the coding tree	Provided in the manuscript
26. Derivation of themes	Derived from the data
27. Software	NA
28. Participant checking	None
Reporting	
29. Quotations presented	Participant quotations presented using participant number and gender
30. Data and findings consistent	Data was consistent as presented
31. Clarity of major themes	Major themes presented in tables and manuscript text
32. Clarity of minor themes	Minor themes are presented and described in tables and manuscript text

Supplementary Figure S2. Example of prompt used for the social AI-enhanced robotic interface (SARI).

Mikael is about to meet his physician for the first time. Mikael is a 68-year-old man who made an appointment a few days back. Apart from the age and sex of the patient, the only information the physician has is that Mikael has sought care because of “ache in the body”.

The following is some information about Mikael’s condition [shortened]:

- He has always been healthy and has not felt any pain similar to that he is seeking for now.
- Sometimes, he experiences back pain, but it has never been present for that long and it has not had the same character.
- In Mikael’s medical charts, it is stated that his blood lipids are above normal.
- Mikael has been advised to eat healthier to avoid developing diabetes and lower his blood lipids.
- Mikael has had a stable blood pressure since he has been on anti-hypertensive treatment.

The following is a dialogue between Mikael and his physician:

- Mikael: Hi doctor.
- Physician: Hello Mikael, my name is Morgan.
- Mikael: Nice to meet you, Morgan. Thanks for seeing me.
- Physician: Of course! Could you describe why you are here today?

Write the next line that Mikael would say.

Supplementary Figure S3. Interview guide exploring students' perception of virtual patient platforms regarding empathetic conduct.

Introduction

- Presentation.
- The purpose of the interview is to explore perceptions and experiences from the usage of virtual patient cases through an AI-driven social robot compared with a conventional semi-linear computer-based platform, for the training of clinical reasoning skills.
- Information about the amount of interviews and recruitment.
- Contact information, neutrality and pseudonymity: Personal information will be saved on locked servers in a coded format prior to analysis. Analysis-ready datasets will be pseudonymised. Results will be reported at an aggregated level.
- Informed consent form (ICF).

Background

- Could you tell me about your background? (i.e. sex, age, previous educational background if any, country of origin, current university)
- Which semester are you attending at the medical programme at this moment and what clinical rotation or course are you attending right now?

Virtual patient cases

- Where were you located when you performed the cases (if not at the clinic)?
- What were your perceptions and thoughts prior to this activity? Did you prepare for the activity in any way?
- Did you experience that you had sufficient time for performing the educational activity? Would you appreciate shorter/longer time allocated?
- How ”close” did you feel towards the patient? Do you experience that this differed across specific cases and/or the two platforms?
- Did you experience that you had the role of a physician during a patient encounter? Please elaborate on your thoughts regarding the patient encounter?
- Which skills did you experience that you were able to practice the most during the virtual patient activities?
- Quality of the patient encounter: Can virtual patient cases be a complement to meeting real-life patients in the clinic for teaching purposes?
- Could you please elaborate what the word clinical reasoning means to you?
- Is there any other instance, or anything else you can think of, where virtual patient simulations can be of benefit if implemented? For example, a formative test, an examination, learning through failure in a safe environment, etc.

Comparisons between the platforms regarding empathy

- Could you please describe your experience with the conventional semi-linear virtual patient simulation platform? Probing question: What aspects of the platform stood out to you in terms of facilitating empathetic conduct?
- Could you please describe your experience with the large language model-based social robotic platform for virtual patient simulations? Probing question: What aspects of the platform stood out to you in terms of facilitating empathetic conduct?
- How would you compare your experience with the two platforms in terms of supporting empathetic behaviour? Please elaborate.
- How do the potential differences impact the overall learning experience? Please elaborate.
- How did you perceive the level of engagement and immersion offered by each platform during virtual patient simulations? Do you believe one platform provides a more realistic and authentic experience? Please elaborate.
- Can you share any specific instances where either one of the virtual patient platforms positively influenced your ability to develop and apply empathetic conduct? How did this differ between the two platforms? Please elaborate.
- Were there any challenges or limitations that you encountered when using either one of the two virtual patient platforms? Please elaborate. Probing question: How did these differ between the two platforms?
- In what ways do you believe the large language model-based virtual patient platform can be improved to better support the development empathetic behaviour? Are there any specific features or functionalities that you would like to see enhanced or added? Please elaborate.
- Did you feel empathic towards the patient? Probing question: please mention aspects from each platform that facilitated this feeling.
- In your opinion, did the level or quality of your self-perceived empathy differ between the two platforms? Please elaborate.
- How do you compare the two platforms to another in terms of fostering empathetic conduct towards a virtual patient?

General comparisons

- Overall, based on your experience with both platforms, which one do you believe holds greater? Please elaborate.

- Overall, do you believe that virtual patient simulations constitute a good educational tool? Please elaborate.

- Overall, do you believe that one or the other platform is more enjoyable in terms of VP-practice. Please elaborate. Probing question: Please mention aspects in which one platform was more enjoyable.

Conclusion

- Do you have anything to add or share in relation to your experience with the virtual patient encounters?
- Do you have anything else to add in general?
- Do you have my contact information?
- Thank you for your participation in the project!

Supplementary Table S4. Examples of the reflexive analysis process.

Transcript	Initial code	Reflexive discussion	**Final code**<br>Theme<br>*(Subtheme)*
"Sometimes when it was worried about something and so on, you still felt that you wanted to answer its worries. And we did it like this several times. We tried to calm down the patient."	**Empathy**	AB and BJ agreed that this segment clearly described spontaneous empathetic feelings toward the VP. Discussions were around emotional or cognitive empathy and the authors concluded that the segment reflects emotional response triggered by perception of the VP as authentic.	**Emotional responses to VPs**<br>Responses to emotional cues<br>*(Responding to concerns)*
"The computer was like a game. And like a text that you read. So I didn't feel close to the patient at all. But for the robot, when it talked, it was still like a real patient."	**Authenticity**	Early in analysis, "authenticity" appeared frequently but with varied meanings. AB and BJ discussed whether this represented technical realism, emotional genuineness, or something else. Through iterative review, they distinguished between perceiving VPs as "like a real patient" (embodied authenticity) and feeling emotionally connected. This resulted in the separation of codes to "perception of patient authenticity" and "presence of emotional connection".	**Perception of patient authenticity**<br>Physical embodiment<br>*(From text to embodied interaction)*
"I'm not an expert in neurology, but if I just try to think about it, it doesn't feel like it's the same parts that are activated by writing a question and formulating it, like actually sitting and formulating it in front of someone. You can't stop halfway through a sentence, erase half of it and rewrite it when you start talking to someone. In a way, you really need to have a good idea of what you're going to say before you say it."	**Communication**<br><br>**Question generation**	Initially coded separately as "Communication" and "Question generation". AB and BJ recognised the overlap between these codes, which both were used to capture the cognitive process of formulating questions in real-time. They agreed that this represented an active cognitive process that was distinct from selecting pre-written options. The code and final theme were refined to frame this notion.	**Differences in question formulation**<br>Cognitive immersion<br>*Active engagement*

VPs: virtual patients.

Supplementary Table S5. Overall Wilcoxon signed-rank test results from VAS data on student preference of VP platforms.

Statistic	Value
N	175
Median (IQR)	2.0 (1.0–4.0)
Mean (SD)	2.6 (± 2.3)
Positive ranks (scores > 5)	13
Negative ranks (scores < 5)	135
Ties (scores = 5)	27
Wilcoxon W	738.5
Z	9.2
P-value	<0.001
Effect size (r)	0.7

Supplementary Table S6. Wilcoxon signed-rank test results from VAS data on student preference of VP platforms based on demographic subgroup: sex.

**Statistic**	**Males**	**Females**
N	84	91
Median (IQR)	2.0 (0.0–4.0)	2.0 (1.0–4.0)
Mean (SD)	2.6 (± 2.3)	2.7 (± 2.2)
Positive ranks (scores > 5)	5	8
Negative ranks (scores < 5)	65	70
Ties (scores = 5)	14	13
Wilcoxon W	172.0	200.0
Z	6.3	6.7
P-value	<0.001	<0.001
Effect size (r)	0.69	0.7

Supplementary Table S7. Wilcoxon signed-rank test results from VAS data on student preference of VP platforms based on demographic subgroup: previous experience.

Statistic	With experience	No experience
N	28	147
Median (IQR)	2.0 (0.0–4.0)	2.0 (1.0–4.0)
Mean (SD)	2.4 (± 2.0)	2.7 (± 2.3)
Positive ranks (scores > 5)	1	12
Negative ranks (scores < 5)	22	113
Ties (scores = 5)	5	22
Wilcoxon W	2.5	617.0
Z	4.1	8.2
P-value	<0.001	<0.001
Effect size (r)	0.78	0.68

Supplementary Table S8. Wilcoxon signed-rank test results from VAS data on student preference of VP platforms based on demographic subgroup: platform order.

Statistic	SARI first	VIC first
N	99	75
Median (IQR)	2.0 (0.0–4.0)	3.0 (1.5–4.5)
Mean (SD)	2.3 (± 2.2)	3.0 (± 2.5)
Positive ranks (scores > 5)	4	8
Negative ranks (scores < 5)	79	56
Ties (scores = 5)	16	11
Wilcoxon W	148.5	217.0
Z	7.3	5.5
P-value	<0.001	<0.001
Effect size (r)	0.73	0.64
